# An Ecological-Developmental Model of Grandparent Caregiving

**DOI:** 10.1093/geroni/igaf122.1642

**Published:** 2025-12-31

**Authors:** Bert Hayslip, Julian Montoro-Rodriguez

**Affiliations:** University of North Texas, Denton, Texas, United States; The University of North Carolina at Charlotte, Charlotte, North Carolina, United States

## Abstract

While a stress and coping as well as a contextual perspective have informed grandparent caregiving, we lack an integrative model that reflects both contextual influences (e.g. service use, neighborhood quality, housing) and developmental change. We present a new and more comprehensive model here stressing the interaction between an evolving social-ecological context (e.g. health vulnerability and service access) and intraindividual developmental change in the grandparent (e.g. late young adulthood, midlife, later life) and the grandchild (e.g. infancy, childhood, adolescence, young adulthood). Key elements of this interactive model, presented with exemplar supportive literature, are a) sociocultural historical shifts defining different cohorts of grandparent caregivers, on par with work identifying grandparent cohort effects, b) emergent uniquenesses of different generations of grandparents, dependent upon when, developmentally, they first become caregivers, be it on-time/age normative or off-time/nonnormative in nature, and c) the impact of caregiving on developmentally significant life domains (e.g., health, work/retirement, grandchild maturation and psychosocial development). How such interrelated influences are navigated will yield different life trajectories for each grandparent cohort, subject to the negative or positive interaction with differential levels of context, e.g. social policy, service access, relationships with age peers, grandchildren, and the biological parent, all of which are shaped by historical change. As grandparent caregiving is countertransitional and whose antecedents are often nonnormative, our developmental-ecological model is grounded in lifespan theory stressing the ongoing interaction of micro-and macro-ecological influences over time, individual differences in grandparent caregiving, and multidimensional developmental outcomes, i.e. health, well-being, mediated by numerous idiosyncratic and historical influences.

